# CD8^+^ T cells in the central nervous system of mice with herpes simplex infection are highly activated and express high levels of CCR5 and CXCR3

**DOI:** 10.1007/s13365-020-00940-2

**Published:** 2021-01-25

**Authors:** Liza Lind, Alexandra Svensson, Karolina Thörn, Malgorzata Krzyzowska, Kristina Eriksson

**Affiliations:** 1 Department of Rheumatology and Inflammation Research, Sahlgrenska Academy, University of Gothenburg, Gothenburg, Sweden; 2 Military Institute of Hygiene and Epidemiology, Kozielska 4, 01-163 Warsaw, Poland

**Keywords:** Central nervous system inflammation, Herpes simplex virus, CD8^+^ T cells, Chemokines

## Abstract

Herpes simplex virus type 2 (HSV-2) is a neurotropic virus that can cause meningitis, an inflammation of the meninges in the central nervous system. T cells are key players in viral clearance, and these cells migrate from peripheral blood into the central nervous system upon infection. Several factors contribute to T cell migration, including the expression of chemokines in the inflamed tissue that attract T cells through their expression of chemokine receptors. Here we investigated CD8^+^ T cell profile in the spinal cord in a mouse model of herpes simplex virus type 2 neuroinflammation. Mice were infected with HSV-2 and sacrificed when showing signs of neuroinflammation. Cells and/or tissue from spinal cord, spleen, and blood were analyzed for expression of activation markers, chemokine receptors, and chemokines. High numbers of CD8^+^ T cells were present in the spinal cord following genital HSV-2-infection. CD8^+^ T cells were highly activated and HSV-2 glycoprotein B -specific effector cells, some of which showed signs of recent degranulation. They also expressed high levels of many chemokine receptors, in particular CCR2, CCR4, CCR5, and CXCR3. Investigating corresponding receptor ligands in spinal cord tissue revealed markedly increased expression of the cognate ligands CCL2, CCL5, CCL8, CCL12, and CXCL10. This study shows that during herpesvirus neuroinflammation anti-viral CD8^+^ T cells accumulate in the CNS. CD8^+^ T cells in the CNS also express chemotactic receptors cognate to the chemotactic gradients in the spinal cord. This indicates that anti-viral CD8^+^ T cells may migrate to infected areas in the spinal cord during herpesvirus neuroinflammation in response to chemotactic gradients.

## Introduction

Herpes simplex virus type 2 (HSV-2) is a double-stranded DNA virus that infects through mucosal tissue to establish a latent infection in the central nervous system (CNS) (Kramer, Cook et al. 2003; Weller and Coen 2012). HSV-2 commonly manifests as genital blisters or remains asymptomatic (Schiffer and Corey 2009). In rare cases HSV-2 can cause meningitis (incidence of 2–4/100 000), an inflammation of the CNS with symptoms such as fever, headaches, and photosensitivity (Bergstrom, Vahlne et al. 1990; Wald and Corey 2007; Kallio-Laine et al. 2009). Although usually benign, HSV-2 meningitis often presents with recurrent episodes of disease (Bergstrom et al. 1990; Kallio-Laine et al. 2009).

T cells are key players in cell-mediated immunity and are reported to be essential in HSV-2 clearance in mice (McDermott et al. 1989; Koelle et al. 1998). A distinct T cell subset are the CD8^+^ cytotoxic T cells, which are involved in direct killing of virus-infected cells by secreting granzymes, inducing target cell apoptosis (Zhang and Bevan 2011). Accumulated evidence implies that CD8^+^ T cells are important not only for the control of acute CNS infection but are also necessary for maintenance of HSV latency in infected ganglia (Schiffer and Corey 2013).

In neuroinflammation T cells migrate from peripheral tissue, crossing the blood-brain barrier to enter CNS tissue and fight the underlying cause. The mechanism of T cell recruitment to the CNS is not fully understood, but it is clear that chemokines, chemotactic cytokines, expressed by cells in the inflamed tissue, are involved (Korn and Kallies 2017). Chemokines bind chemokine receptors, which are G protein-linked transmembrane receptors expressed on all immune cells, but expression differs between cell types, activation states and tissues (Melik-Parsadaniantz and Rostene 2008; Griffith et al. 2014). CXCR3 and its ligand CXCL10 are known to have protective roles in HSV-2 infection as well as to facilitate migration into the CNS (Thapa et al. 2008; Thapa and Carr 2009). Chemokines CCL3, CCL5, and CXCL2 have been implicated in enhanced viral clearance and/or survivability (Eo et al. 2001; Harle et al. 2001; Benencia et al. 2003), and in humans chemokines have been characterized in the acute phase of HSV-2 meningitis (Lind et al. 2017). However, the chemokine-receptor profile of CD8^+^ T cells transmigrating to the CNS in HSV-2 meningitis is not known.

The present study was initiated to elucidate chemokine and chemokine receptor expression affecting CD8^+^ T cell migration into the CNS of HSV-2-infected mice. Using a mouse model of HSV-2-induced neuroinflammation, we show that effector CD8^+^ T cells express a chemokine repertoire that suggests that they have migrated to the CNS. These cells are highly activated, granzyme B-expressing cells with high specificity for HSV-2 glycoprotein B. In the CNS, the CD8^+^ T cells express a wide range of chemokine receptors; CCR2, CCR4, CCR5, CXCR3, and also CXCR4. Several ligands of these receptors, especially CCL2, CCL5, CCL8, and CXCL10, were highly upregulated in the CNS of mice with HSV-2-induced neuroinflammation. Our data indicate that CD8^+^ T cells migrate to and accumulate in the CNS, where they exert their function to battle HSV-2.

## Materials and methods

### Infection

About 12–20-week-old female C57bl/6 mice (Charles River) were infected with HSV-2 strain 333 as previously described (Svensson et al. 2005). In short, mice were subcutaneously injected with 2-mg medroxyprogesterone acetate (Depo-Provera, Pfizer). Six days post treatment mice were infected with 40 000 PFU HSV-2 strain 333 intravaginal and sacrificed 2, 4, 6, or 8 days post infection. Control mice were left uninfected. Mice were monitored and scored according to the following: 0, no signs of infection/inflammation; 1, mild genital redness; 2, moderate genital inflammation; 3, obvious damage of the genital mucosa, impaired general condition; and 4, hind leg paralysis (neuroinflammation). The numbers of mice used for each experiment were as follows: flow cytometry for activation markers, *n* = 5 mice/group; flow cytometry for chemokine receptor expression, *n* = 4 mice/group and *n* = 15 mice/group at day 0 and day 8, respectively; and chemokine and cytokine expression in spinal cord, *n* = 10 and *n* = 11 at days 0 and 8, respectively. Mice were kept under pathogen-free conditions at the Department of Rheumatology and Inflammation Research, University of Gothenburg (Gothenburg, Sweden). This study was approved by the ethical committee for animal experiments (Gothenburg, Sweden).

### Confocal imaging

The spinal cord was fixed in 4% paraformaldehyde, then saturated with 30% sucrose, frozen in liquid nitrogen, and cut into 15-µm thick cryostat sections. Sections were washed with PBS and pre-incubated with 3% BSA (Sigma-Aldrich) diluted in 0.1% saponin (Sigma-Aldrich) in PBS for 1.5 h at room temperature. When using mouse monoclonal antibody, Mouse on Mouse (M.O.M.®) blocking reagent kit (Vector Laboratories, Peterborough, UK) was applied for pre-incubation. Sections were then incubated overnight at 4 °C with primary antibodies diluted 1:200 in working solution (2% BSA, 0.1% saponin in PBS). Antibodies used included rabbit polyclonal anti-HSV-1/2 (cat. # B0114; Dako, Agilent, Santa Clara, CA, USA), mouse monoclonal anti-GFAP (clone 2A5) (cat. # ab4648, Abcam, Cambridge, UK), and rat anti-CD8-biotin (clone 53-6.7) (cat. # 553028, BD). Biotinylated Abs were detected with Alexa Fluor™ 555 Tyramide SuperBoost™ Kit (ThermoFisher Scientific), while other primary antibodies were detected using Alexa Fluor 647 anti-mouse (cat. # A-21235) or Alexa Fluor 488 anti-rabbit (cat. # A31572) polyclonal antibodies (ThermoFisher Scientific). After final washing in PBS, slides were closed in SlowFade™ Diamond Antifade Mountant with 4-6-diamidino-2-phenylindole (DAPI; ThermoFisher Scientific). Sections were imaged by confocal microscopy using a Zeiss Laser Scanning Inverted Microscope LSM-700 equipped with 40X/1.3 Oil NA objective and Black Zen software (Carl Zeiss) at the Center for Cellular Imaging, University of Gothenburg.

### Flow cytometry

Spleens and spinal cords were collected and mashed through a 70-µM cell strainer. Spleen red blood cells were lysed using ammonium chloride. Cells were counted using an automated hematology analyzer (KX-21N, Sysmex), and 10^6^ million cells/sample were subsequently used for flow cytometric staining. Spinal cords were resuspended in 2 ml PBS, and 200 µL were used for staining one sample. After blocking of unspecific binding for 10 min using 5 µg/mL rat anti-mouse CD16/CD32 (cat no 553142), cells were stained for 20 min using the following mAbs, CD44 PE (cat no 561860), CD8 BV421 (cat no 563898), CCR7 PerCp-Cy5.5 (cat no 560812) (BD Biosciences), CCR2 PE (cat no FAB5538P), CXCR3 PE (cat no FAB1685P), CXCR4 FITC (cat no FAB21651F) (R&D Systems), CCR4 APC (cat no 131211), CCR4 PE (cat no 131203), CCR5 APC (cat no 107011), CCR6 PerCp-Cy5.5 (cat no 129810), CXCR3 FITC (cat no 126535), CXCR4 APC (cat no 146507), CD107a PerCP-Cy5.5 (cat no 121625), CD3 FITC (cat no 100306), (Biolegend), CD62L APC (cat no 17–0621-82, eBioscience), and SSIEFARL APC (ProImmune). For intracellular staining, BD cytofix/cytoperm fixation/permeabilization kit (BD) was used according to manufacturer’s instructions. Intracellular staining was done using FITC rat anti-granzyme B (cat no 15208379, eBioscience) and PE rabbit anti-active caspase-3 (cat no 561011 BD). Cell viability was assessed by staining with Fixable Viability Dye eFluor 506 (cat no 65-0866-14, eBioscience) (Suppl. Fig. 1). Stained cells were acquired using BD FacsVerse (BD Biosciences) and analyzed using FlowJo software (Tree Star, Ashland, OR, USA). Fluorescence minus one was used as control for cell-surface staining and isotype controls were used for intracellular staining.

**Fig. 1:**
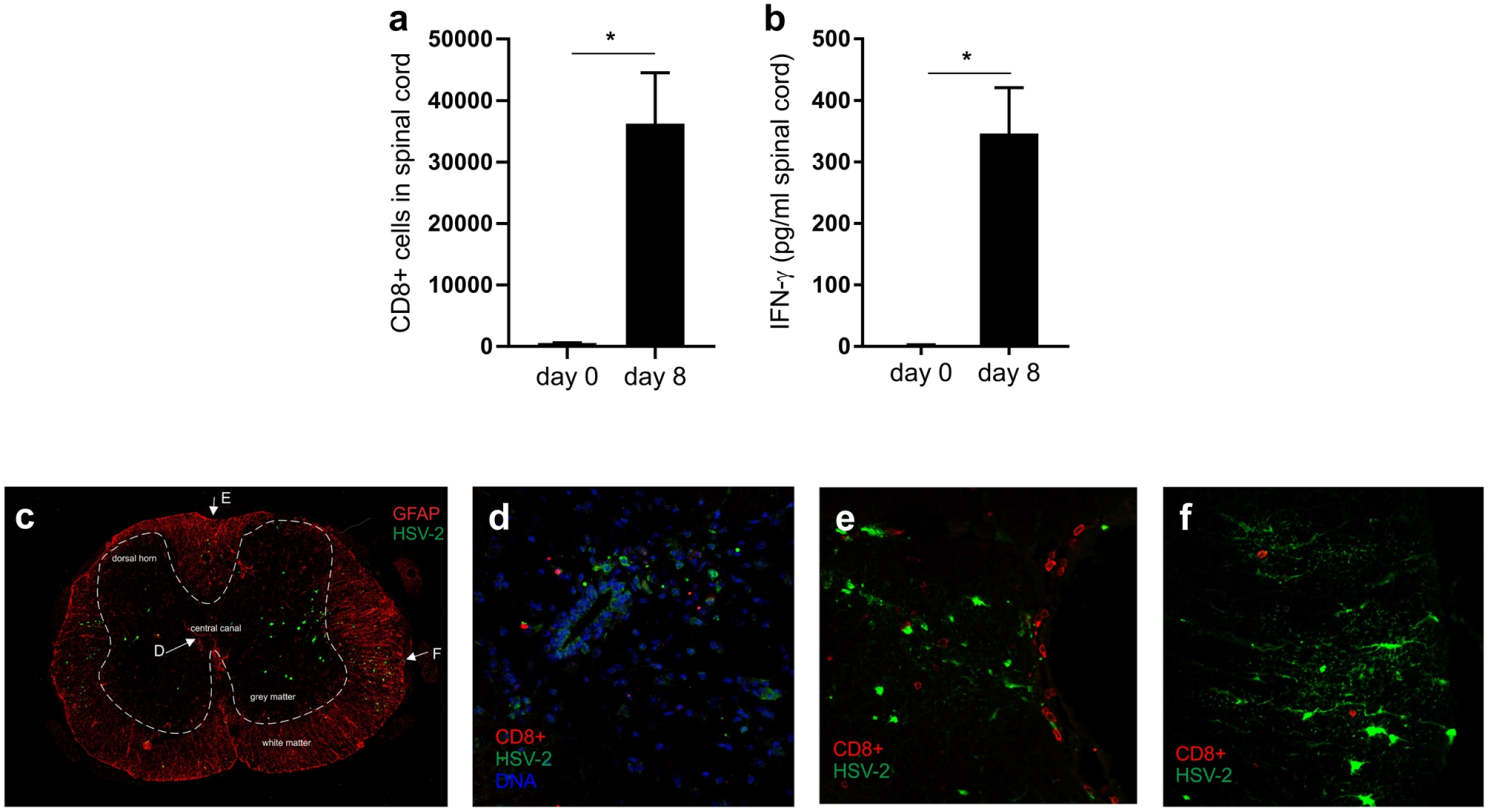
CD8^+^ T cells in spinal cord from healthy and HSV-2 infected mice. **a** Total number of CD8^+^ T cells and **b** IFN-gamma levels (*n* = 10) day 0 and day 8 in the spinal cord of HSV-2-infected mice. Data are presented as mean ± SEM using unpaired *T* test where the asterisk indicates *p* < 0.05. Statistical analysis was performed using GraphPad Prism version 7 (GraphPad Software). **c-f** CD8^+^ T cells in HSV-2 infected spinal cord at 8 days p.i.; **c** transverse section through the spinal cord in which white matter was distinguished by anti-GFAP staining (the marker for astrocytes, red). CD8^+^ T cells (red) around HSV-2 infected cells (green) were identified mainly in the area of **d** central canal in grey matter, **e** dorsal medial sulcus, and **f** lateral funiculus in white matter

### Chemokine and cytokine analysis

Whole blood was collected and processed into serum by allowing clotting in room temperature, followed by separation of the serum phase by centrifugation. Extractions of tissue was achieved by collecting spinal cords in PBS with 0.1 mg/ml soybean trypsin inhibitor (Sigma), 1.5 mM Pefabloc (Coatech AB), 50 mM EDTA, and 0.1% bovine serum albumin. Samples were weighed and frozen. Upon thawing, samples were permeabilized using 2% saponin over night at 4 °C, and the supernatant was subsequently collected by centrifugation. Chemokine and cytokine analysis was performed using a mouse magnetic Luminex assay (CCL2 (lowest level of quantification (LLOQ) = 415 pg/ml), CCL3 (LLOQ = 11 pg/ml), CCL4 (LLOQ = 288 pg/ml), CCL7 (LLOQ = 8 pg/ml), CCL8 (LLOQ = 8 pg/ml), CCL11 (LLOQ = 19 pg/ml), CCL12 (LLOQ = 8 pg/ml), CCL20 (LLOQ = 216 pg/ml), CCL22 (LLOQ = 29 pg/ml), CXCL1 (LLOQ = 72 pg/ml), CXCL2 (LLOQ = 6 pg/ml), CXCL5 (LLOQ = 63 pg/ml), CXCL10 (LLOQ = 153 pg/ml), CXCL12 (LLOQ = 525 pg/ml), GM-CSF (LLOQ = 21 pg/ml), IFN-gamma (LLOQ = 20 pg/ml), IL-1alpha (LLOQ = 63 pg/ml), IL-1beta (LLOQ = 302 pg/ml), IL-2 (LLOQ = 11 pg/ml), IL-4 (LLOQ = 87 pg/ml), IL-6 (LLOQ = 57 pg/ml), IL-10 (LLOQ = 35 pg/ml), TNF-alpha (LLOQ = 3 pg/ml)) from R&D Systems according to manufacturer’s instructions and analyzed using Bio-Plex 200 system (Bio-Rad). The levels of CCL5 were assessed using a DuoSet ELISA kit (R&D, Minneapolis, MN, USA, LLOQ 31 pg/ml) according to manufacturer’s instruction.

### Statistical analysis

All data are expressed as mean ± SEM. Unpaired *T* test was used when two groups were compared, and one-way ANOVA with Dunnett’s or Tukey´s multiple comparisons test was used when three or more groups were compared. Statistical analysis was performed using GraphPad Prism version 7 (GraphPad Software).

## Results

### Increase of CD8^+^ T cells in the spinal cord upon HSV-2 infection

To investigate the CD8^+^ T cell profile in the CNS, mice were infected with HSV-2. Neurological symptoms were observed 7–9 days post infection (i.e., hind leg paralysis). Animals were then euthanized, and cells from spinal cord (CNS tissue) and spleen (peripheral tissue) were used for flow cytometric analysis both before and after infection. Low numbers of CD8^+^ T cells were detected in spinal cord from healthy animals. Following HSV-2 infection the numbers increased almost 100-fold, compared with healthy animals (Fig. 1a, Suppl. Fig. 1). This was associated with elevated levels of IFN-gamma in spinal cord extracts (Fig. 1b). The CD8^+^ T cells that accumulated near the virus were found in both grey and white matter, mainly in the area of the central canal (Fig. 1d), the dorsal medial sulcus (Fig. 1e), and the lateral funiculus (Fig. 1f). Parts of the spinal cord CD8^+^ T cells had entered into the apoptotic pathway and were either pre-apoptotic (viable cells expressing caspase-3), apoptotic (non-viable cells expressing caspase-3), or dead (Suppl. Fig. 1e). Thus, there is a vast increase of CD8^+^ T cells in the spinal cord during herpes simplex infection, indicating that CD8^+^ T cells traffic into the CNS during viral neuroinflammation.

###  CD8^+^ T cells in the spinal cord of HSV-2-infected mice have a highly activated HSV-2 specific phenotype

**Fig. 2:**
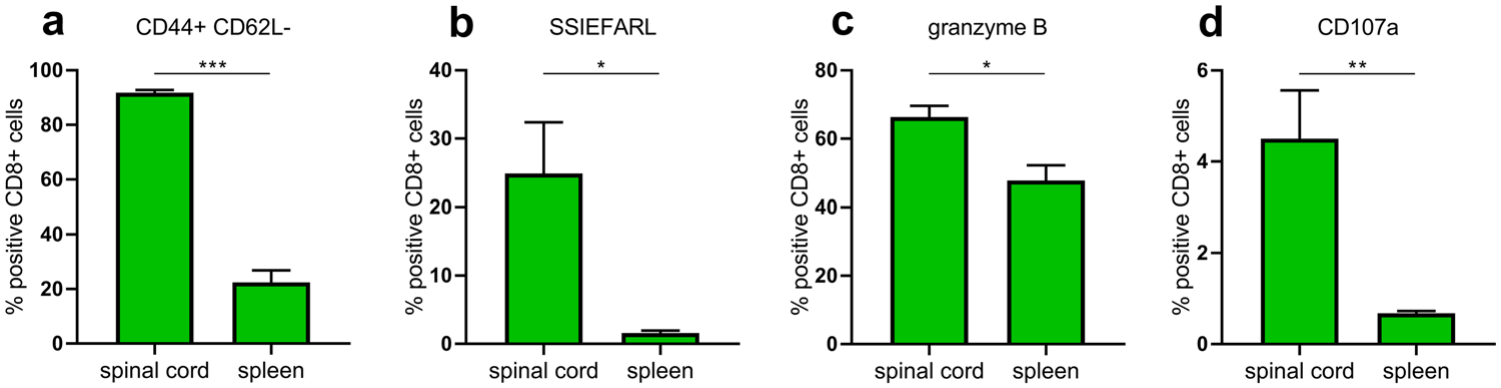
Activation marker expression on CD8^+^ T cells in spinal cord and spleen of HSV-2-infected mice. **a** Activation (defined as CD44^+^CD62L^−^ cells), **b** HSV-2 specificity to SSIEFARL peptide, **c** granzyme B expression, and **d** degranulation (defined as CD107a^+^ cells) of CD8^+^ T cell populations in spleen and spinal cord day 8 post HSV-2 infection (*n* = 5/group). Data are presented as mean ± SEM. Statistical analysis was performed using unpaired *T* test, where *** indicates *p* < 0.001, ** *p* < 0.01 and * *p* < 0.05 using GraphPad Prism version 7 (GraphPad Software)

To prove the hypothesis that CD8^+^ T cells found in the CNS are virus-specific effector cells, we characterized the viable CD8^+^ T cells by flow cytometry (Suppl. Fig. 2). In spinal cord almost all CD8^+^ T cells were highly activated, defined as CD44+ CD62L−, while CD8^+^ T cells in the spleen showed a significantly lower activation frequency (Fig. 2a). Staining cells with the HSV-2 specific MHC class 1-pentamer SSIEFARL showed that a 15-fold higher proportion of CD8^+^ T cells were specific for SSIEFARL in spinal cord compared with spleen (Fig. 2b). Most CD8^+^ T cells in the spinal cord expressed the cytotoxic enzyme granzyme B (Fig. 2c), and approximately 4% of the cells showed signs of recent degranulation as indicated by the marker CD107a (Fig. 2d). In summary, CD8^+^ T cells present in the spinal cord during herpes simplex induced neuroinflammation are highly activated virus-specific effector T cells.

### Chemokine receptor expression in CD8^+^ T cells in the spinal cord of herpes simplex induced neuroinflammation

To assess contributing factors of T cell presence in the CNS upon HSV-2 infection, chemokine receptors on the CD8^+^ T cells from mice 8 days post infection were analyzed by flow cytometry and compared with the levels in uninfected mice and to the levels on spleen CD8^+^ T cells (Suppl. Figs. 3 and 4).

**Fig. 3:**
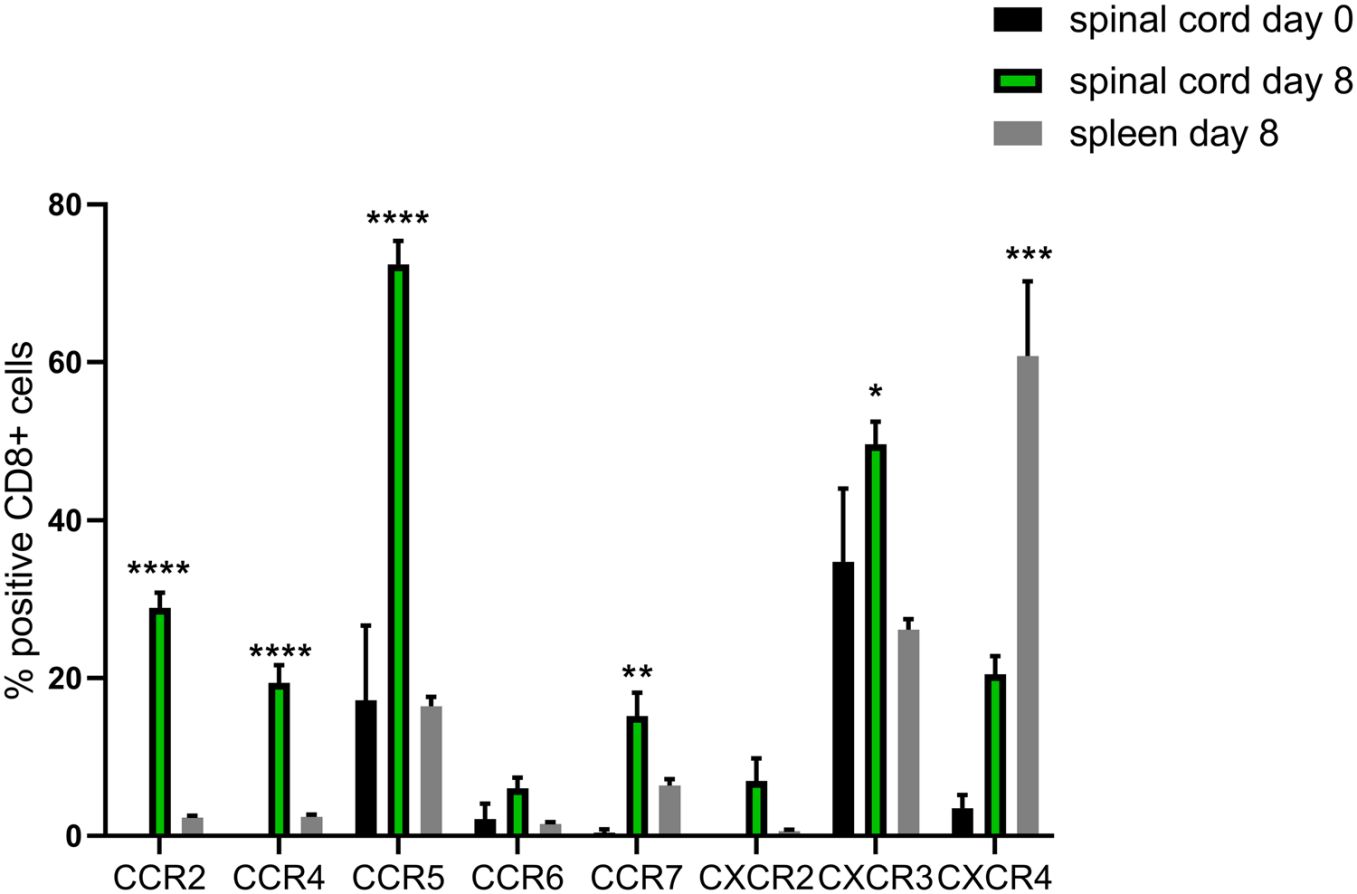
Chemokine receptor expression on CD8^+^ T cells in spinal cord and spleen of HSV-2-infected mice. Data are presented as mean ± SEM. Black bars represent spinal cord cells day 0 (*n* = 4), green bars represent spinal cord cells day 8 (*n* = 15), and grey bars represent spleen cells day 8 (*n* = 15) post HSV-2 infection. Statistical analysis was performed using ANOVA with Dunnett’s multiple comparisons test, where **** indicates *p* < 0.0001; *** *p* < 0.001; ** *p* < 0.01, and * *p* < 0.05 using GraphPad Prism version 7 (GraphPad Software). Statistics refer to the comparison of spinal cord cells d8 vs spinal cord cells d0 and spleen cells d8 (CCR2, CCR4, CCR5, CCR7, and CXCR3); and spinal cord cells d8 vs spleen cells d8 (CXCR4)

**Fig. 4:**
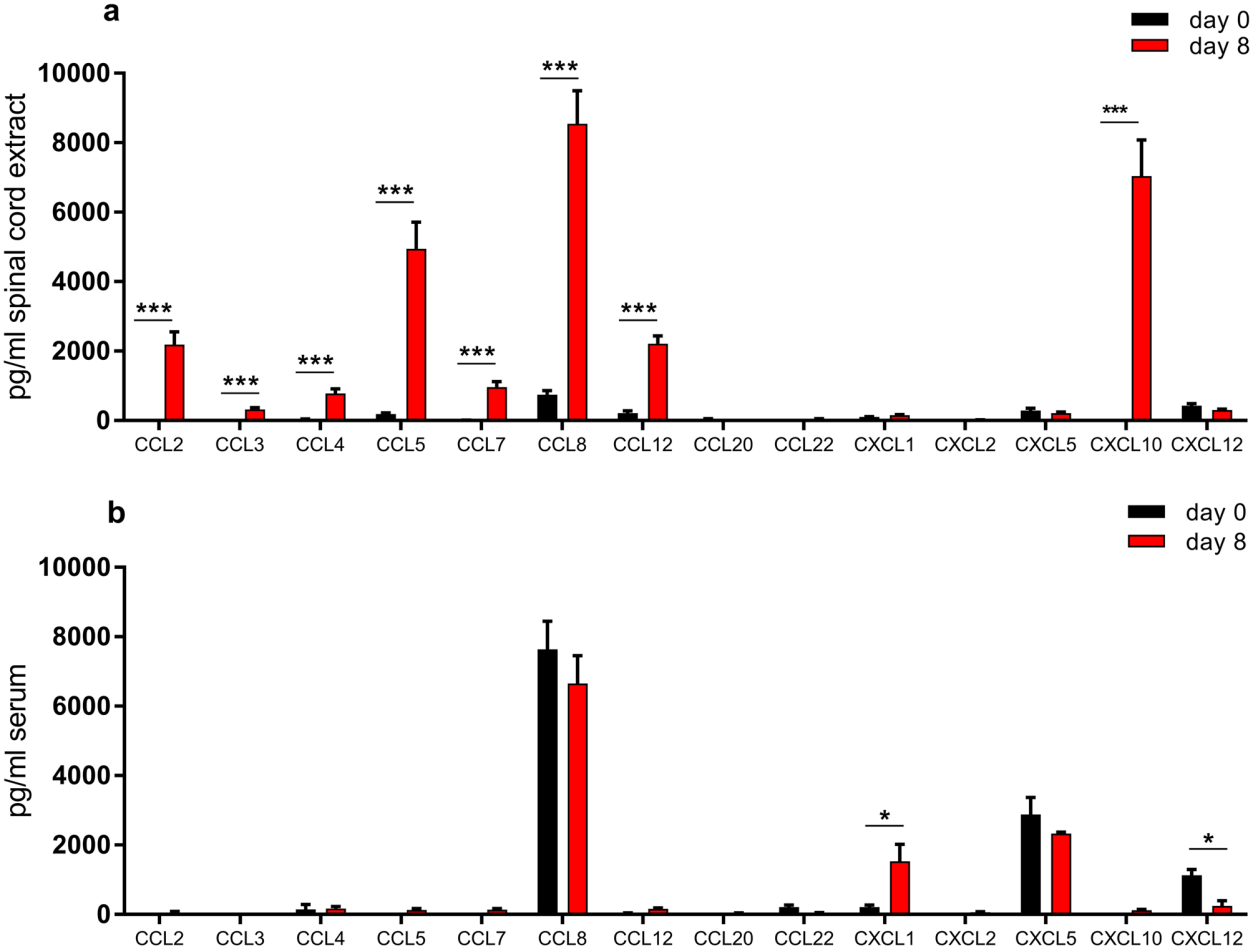
Chemokine expression in spinal cord and sera of HSV-2 infected mice. Chemokine levels in **a** spinal cord extracts and **b** sera of mice infected with HSV-2 were measured at 0 (*n* = 10) or 8 (*n* = 11) days post infection. Data are presented as mean ± SEM using unpaired T test where *** indicates *p* < 0.001, ** *p* < 0.01, and * *p* < 0.05 using GraphPad Prism version 7 (GraphPad Software)

CD8^+^ T cells in spinal cord from uninfected mice had a restricted chemokine receptor repertoire with 20% of the cells expressing CCR5 and 40% expressing CXCR3 (Fig. 3, black bars). The CD8^+^ T cells that appeared in the spinal cord after infection had a more diverse chemokine receptor expression pattern compared with both CD8^+^ T cells in the spinal cord of uninfected mice (Fig. 3, green bars) and to CD8^+^ T cells in the spleen of HSV-2 infected animals (Fig. 3, grey bars), and there were also higher frequencies of chemokine-receptor-positive CD8^+^ T cells (Fig. 3). The majority of CD8^+^ T cells in the spinal cord of HSV-2 infected mice expressed CCR5 and CXCR3 (Fig. 3, green bars). The frequencies of CD8^+^ T cells expressing CCR2, CCR4 or CCR7 were also higher in the spinal cord in HSV-2-infected mice compared with spinal cord CD8^+^ T cells in uninfected mice and also compared with CD8^+^ T cells in spleen from infected animals, with frequencies in the range of 20–30% of CD8^+^ T cells. CCR7 was also more frequently expressed on spleen CD8^+^ T cells from uninfected mice compared with spleen CD8^+^ T cells from infected mice (Suppl. Fig. 5). CXCR4 on the other hand was expressed by a majority of spleen CD8^+^ T cells, less so by CD8^+^ T cells in spinal cord from infected mice, and hardly at all by spinal cord and spleen CD8^+^ T cells from uninfected mice (Fig. 3, Suppl. Fig. 5). Taken together, these data show that CD8^+^ T cells present in the CNS express CCR5 and CXCR3 during HSV-2-induced neuroinflammation.

### Chemokine expression pattern in the spinal cord of mice with HSV-2-induced neuroinflammation

We used multiplex analysis of spinal cord tissue extractions to evaluate if cognate ligands to CCR2, CCR4, CCR5, CXCR3, and CXCR4 are induced in the spinal cord of mice upon HSV-2 infection, and also compared this with the levels induced in serum upon HSV-2 infection. There was a strong induction of CCL5 and CCL8 (which are ligands to CCR5) and CXCL10 (which is a CXCR3 ligand) in the spinal cord of HSV-2-infected mice (Fig. 4a). Also, CCL2 (ligand for CCR2 and CCR4), CCL3 (ligand for CCR4 and CCR5), CCL7, and CCL12 (ligands for CCR2) showed a moderate but significant increase in spinal cord at day 8 compared with uninfected mice (day 0), while CCL4, CCL20, CCL22, CXCL1, CXCL2, CXCL5, and CXCL12 remained low.

In blood, CCL8 and CXCL5 dominated in healthy animals, but CXCL12 was also detected (Fig. 4b). Upon HSV-2 infection, the serum levels of CCL8 and CXCL12 decreased slightly while CXCL1 was increased (Fig. 4b).

## Discussion

In this study we show that highly activated virus-specific CD8^+^ T cells expressing CCR5 and CXCR3 are found in the spinal cord of HSV-2-infected mice. This was associated with elevated levels of cognate chemokines in the spinal cord during HSV-2 infection.

We confirm previous studies showing that there are few CD8 + T cells in the spinal cord of naïve mice (Deb and Howe 2009). For the first time though, we can show that the majority of these CD8^+^ T cells have a limited expression of chemokine receptors. About 20–30% of the cells expressed CCR5 and/or CXCR3 and few/none expressed any of the other chemokine-receptors investigated: CCR2, CCR4, CCR6, CCR7, CXCR2, or CXCR4. In accordance, also, the chemokine levels in the spinal cord were low in the absence of infection, as previously documented both in humans and in mice (Thapa et al. 2008; Lind et al. 2017). Thus, a limited number of CD8^+^ T cells may patrol the healthy spinal cord in what appear to be a chemokine-independent fashion.

Neuroinflammation caused by HSV-2 infection led to a pronounced increase of CD8^+^ T cells in the spinal cord, as shown by us here and by others (Thapa et al. 2008). The viable fraction of the spinal cord CD8^+^ T cells in HSV-2 infected mice was CD44+ (and CD62L−) effector/memory cells, similar to what has been shown in other inflammatory diseases of the CNS (Coque et al. 2019) and in cell adhesion and migration to the CNS in general (Brocke et al. 1999). The majority of the cells expressed granzyme B, and a few percent of them had recently degranulated. As previously shown (Thapa et al. 2008), CD8^+^ T cells that were specific for the HSV-2 peptide SSIEFARL predominated in the spinal cord of HSV-2-infected mice, in accordance with the notion that only antigen-specific effector cells translocate to the CNS. We also found that a fraction of the CD8^+^ T cells in the spinal cord of HSV-2 infected mice had entered into an apoptotic pathway. The antiviral activity in the brain is tightly controlled, and high doses of HSV-1 in the mouse brain have been shown to induce apoptosis of immune cells (Reinert et al. 2020). We conclude that HSV-2-induced neuroinflammation in mice is associated with the presence of highly activated virus-specific CD8^+^ T cells in the spinal cord.

The majority of the spinal cord CD8^+^ T cells expressed CCR5 and several CCR5 ligands were expressed in the HSV-2-infected spinal cord. Studies in gene-deficient mice show that albeit CCR5 deficiency is associated with exacerbated CNS disease, it does not affect CD8^+^ T cell numbers in the CNS, neither during HSV-2 infection nor during intracranial infection with coronavirus (Glass and Lane 2003; Thapa et al. 2007). This is in contrast to studies on non-viral CNS inflammation where CCR5 deficiency leads to an attenuated influx of CD8^+^ T cells and other immune cells to the spinal cord (Badell et al. 2015; Gu et al. 2016). Many ligands for CCR5 are however expressed in the HSV-2-infected spinal cord. Thapa et al. have previously reported the presence of CCL3 and CCL5 (Thapa et al. 2007; Thapa et al. 2008; Thapa and Carr 2009), and in this study we show that also CCL4 and in particular CCL8 are upregulated. The primary binding partner of CCL8 is CCR5, which is expressed on activated T cells (Mack et al. 2001) as confirmed by us. The latter is particularly interesting as CCL8 is one of the most prominent chemokines in HSV-2 meningitis patients (Lind et al. 2017). We conclude that CCR5 is present on most CD8^+^ T cells in the spinal cord during viral neuroinflammation, with concurrent high levels of CCR5 ligands including in particular CCL8.

Almost 60% of the CD8^+^ T cells in the spinal cord of HSV-2-infected mice also expressed CXCR3, and the cognate ligand CXCL10 was markedly upregulated in the infected spinal cord. CXCR3 is an important chemokine receptor for T cell migration; however, it is debated if CXCR3 has a protective or detrimental function. On one hand, mice deficient in CXCR3 succumb faster to HSV-2 infection; on the other hand, HSV-1-infected mice lacking CXCR3 are protected from fatal encephalitis (Thapa and Carr 2009; Zimmermann et al. 2017), indicating a mouse strain and tissue-specific role of CXCR3 (Lundberg et al. 2007). Lack of the CXCR3 ligands CXCL9 and CXCL10 on the other hand increase the susceptibility to HSV-2 infection in mice and reduce T cell migration into the spinal cord despite high levels of CCL2, CCL3, and CCL5 (Thapa et al. 2008), implying that CXCR3/CXCL10 represent a major pathway for spinal cord T cell recruitment. IFN-gamma, which we could show is expressed in the infected spinal cord, induces the expression of CXCL10. CXCL10 is also upregulated in other neuroinflammatory infections like Theiler’s murine encephalomyelitis virus, lymphocytic choriomeningitis virus, and tick-borne encephalitis (Hoffman et al. 1999; Nansen et al. 2000; Pokorna Formanova et al. 2019). Most importantly, the enhanced levels of CXCL10 correlate with data from cerebrospinal fluid from humans with HSV-2 meningitis (Lind, Studahl et al. 2017).

A moderate proportion of CD8^+^ T cells in the infected spinal cord also expressed CCR2 and CCR4. CCR2, usually considered a monocyte marker, is found on 2–15% of T cells in both healthy humans and mice, where it binds ligands CCL2, CCL7, CCL8, and CCL12 (Mack et al. 2001; Kivisakk et al. 2003; Semple et al. 2010). These four chemokines were strongly upregulated in the spinal cord of mice with HSV-2-induced neuroinflammation, as well as in human HSV-2 meningitis patients (Lind, Studahl et al. 2017). Continuing the chain of promiscuous chemokine receptor/ligand interactions CCL5, together with CCL2 and CCL3, also interacts with CCR4. This chemokine receptor is normally considered a classical T_H_2 and Treg marker (Morimoto et al. 2005; Molinaro et al. 2015). In humans it is also expressed on a subset of cytokine-producing but non-cytotoxic memory CD8^+^ T cells (Kondo and Takiguchi 2009), which resemble a CD8^+^ T cells subset that are involved in the inhibition of HSV reactivation from latency (Kondo and Takiguchi 2009). In summary, all measured chemokines binding either CCR2 or CCR4 are increased in the spinal cord of HSV-2-infected mice, possibly contributing to the recruitment of CCR2- and CCR4-expressing CD8^+^ T cells.

CXCR4 was found on a limited number of spinal cord CD8^+^ T cells but was abundantly expressed on T cells in the spleen of HSV-2-infected mice, indicating an inverse relationship to CNS homing. CXCR4 interacts with CXCL12, which is a known retention signal for CD8^+^ T cells at the blood–brain barrier. In West Nile virus infection it has been reported that disturbing the CXCR4/CXCL12 interaction allows migration of CD8^+^ T cells into the brain parenchyma resulting in increased viral clearance and survival (McCandless et al. 2008; Michlmayr and Lim 2014). In HSV-2 infection, the CXCL12 levels remained low in spinal cord confirming that the CXCR4/CXCL12 pathway is not a major driving force for CD8^+^ T cell presence in the spinal cord.

In conclusion, we have characterized the chemokine receptor expression pattern on CD8^+^ T cells in spinal cord during steady state and upon HSV-2 neuroinflammation. During homeostasis the CD8^+^ T cells that patrol the spinal cord express few chemokine receptors. Upon HSV-2 infection there is a pronounced increase of CCR5- and CXCR3-expressing highly activated virus-specific CD8^+^ T cells. These data agree with the chemokine secretion pattern in the spinal cord with particularly high levels of CCL5, CCL8, and CXCL10. Importantly, the high levels of CXCL10 correspond to the levels found in cerebrospinal fluid from humans with HSV-2 meningitis. The role of chemokine receptor profile and chemokines in CD8^+^ T cell trafficking to the CNS should be further investigated to evaluate the contribution to disease severity and/or clearance of the infection, and as a potential target for new treatments.

## Supplementary Information

Below is the link to the electronic supplementary material.

Supplementary file1 (PDF 627 KB)
