# Supplementary material for: CD8+ T cells in the central nervous system of mice with herpes simplex infection are highly activated and express high levels of CCR5 and CXCR3
Source: J Neurovirol. 2021 Jan 25;27(1):145–53. doi: 10.1007/s13365-020-00940-2 (PMC7831625; doi:10.1007/s13365-020-00940-2)

Supplementary figure 1. Representative flow cytometry plots of spinal cord cells showing the general gating strategy for staining of spinal cord and spleen cells Plots show (A) SSC-A (y-axis) vs FSC-A (x-axis), (B) FCS-H (y-axis) vs FSC-A (x-axis), (C) CD3 (y-axis) vs FSC-A (x-axis), (D) CD8 (y-axis) vs CD3 (x-axis), and (E) live (green) and dead/apoptotic (red) CD8+ T-cells after staining with Caspase-3 (y-axis) and Viability Dye (x-axis).

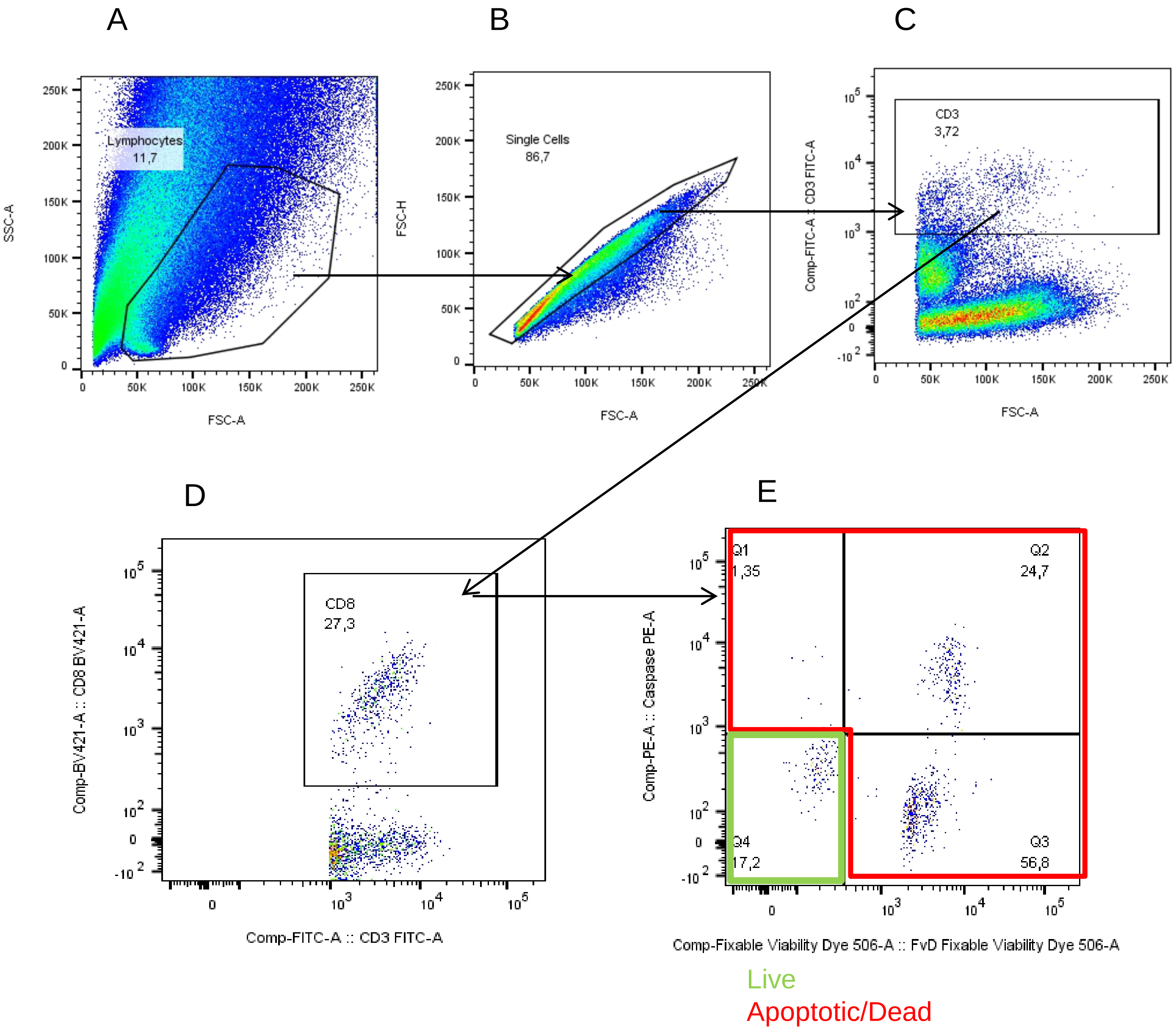

Supplementary figure 2. Representative flow cytometry plots of spleen cells showing the gating strategy for staining of spleen and spinal cord CD8+ T-cells with (A) CD44 and CD62, (B) SSIEFARL, and (C) and CD107a.

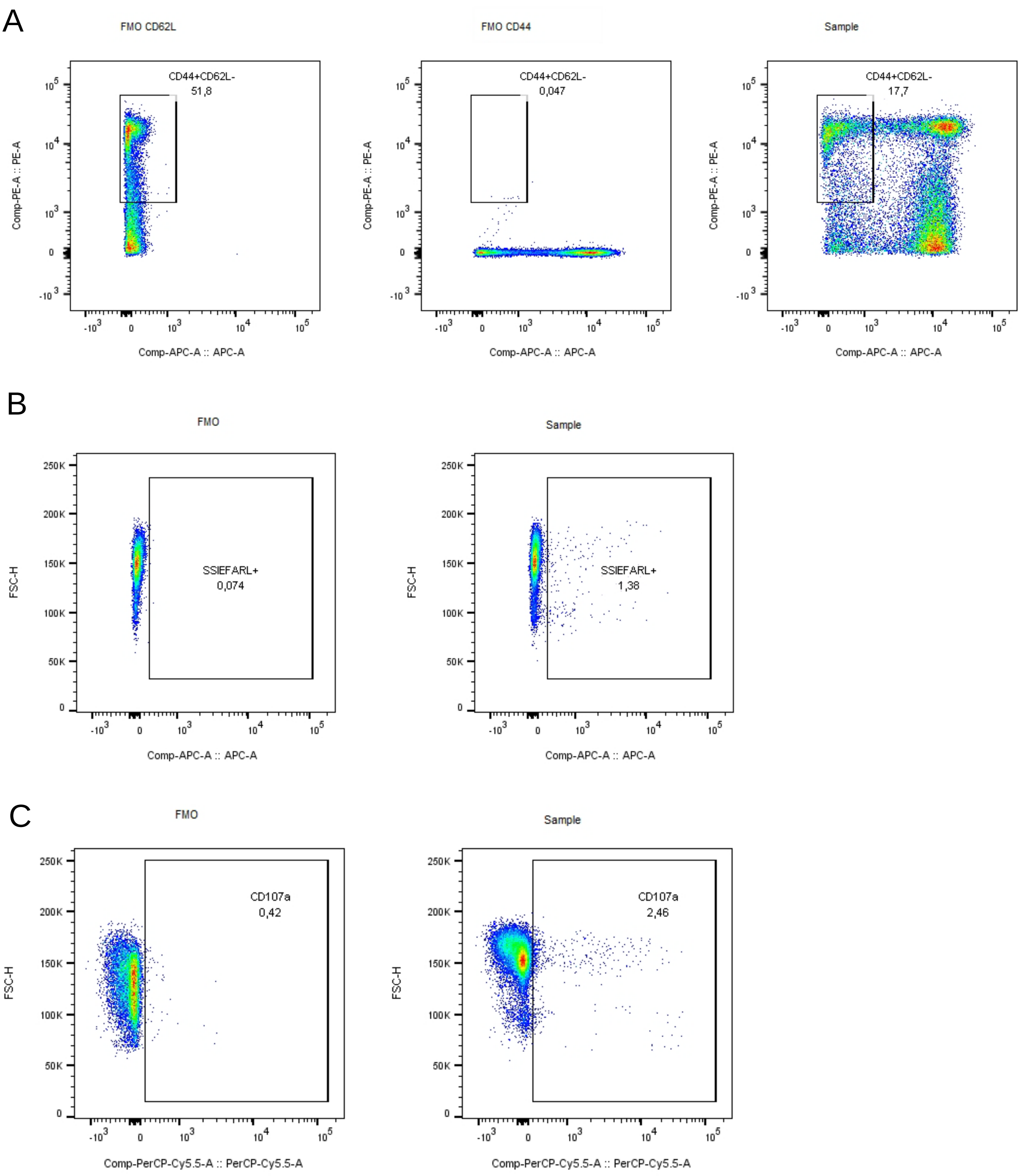

Supplementary figure 3. Representative flow cytometry plots of spleen cells showing the gating strategy for staining of spleen and spinal cord CD8+ T-cells with CCR2 (A, right panel), CCR4 (B, right panel), CCR5 (C, right panel), and CCR6 (D, right panel). Left panel represent FMO staining for the corresponding chemokine receptor.

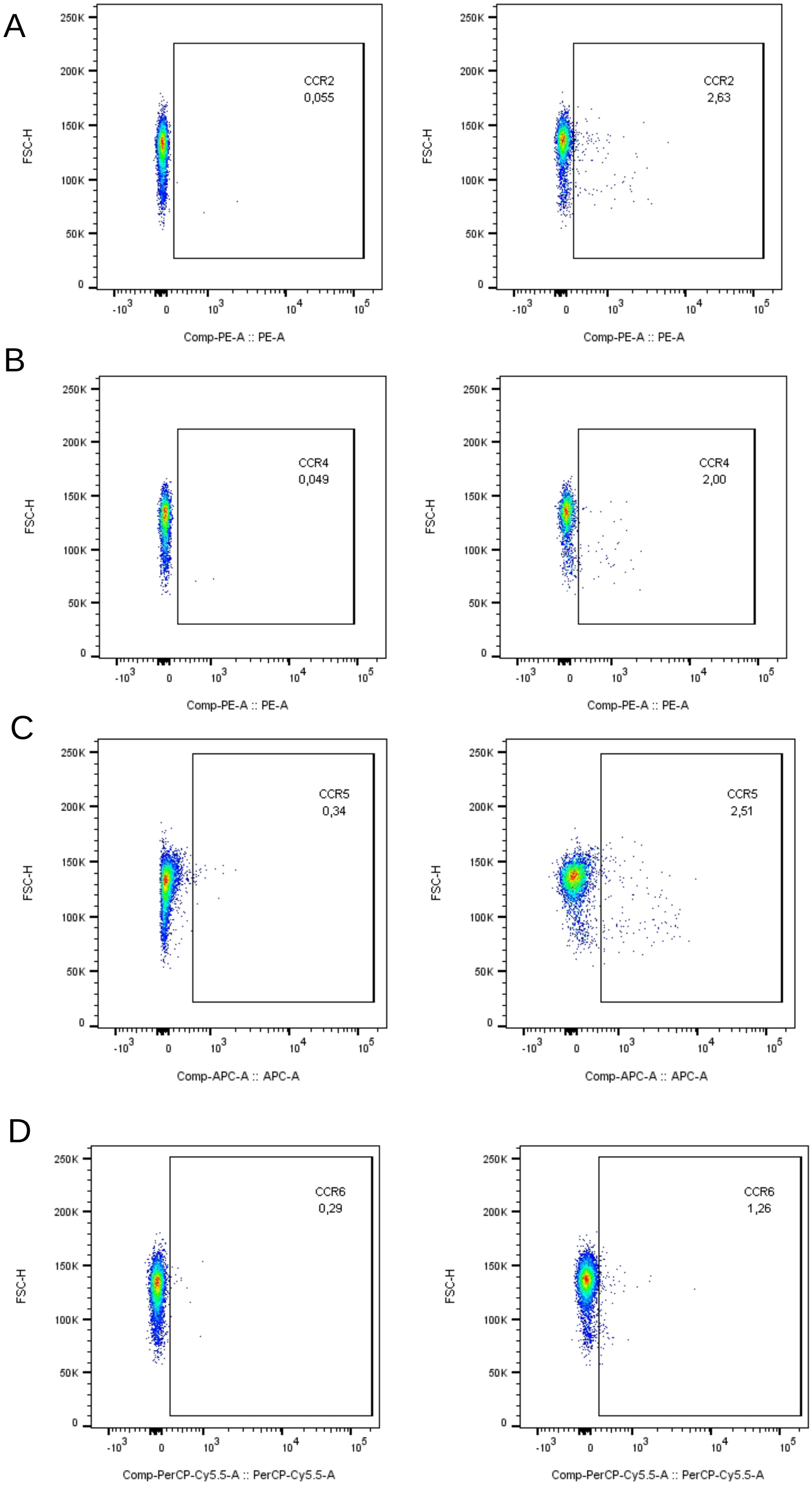

Supplementary figure 4. Representative flow cytometry plots of spleen cells showing the gating strategy for staining of spleen and spinal cord CD8+ T-cells with CCR7 (A, right panel), CXCR2 (B, right panel), CXCR3 (C, right panel), and CXCR4 (D, right panel). Left panel represent FMO staining for the corresponding chemokine receptor.

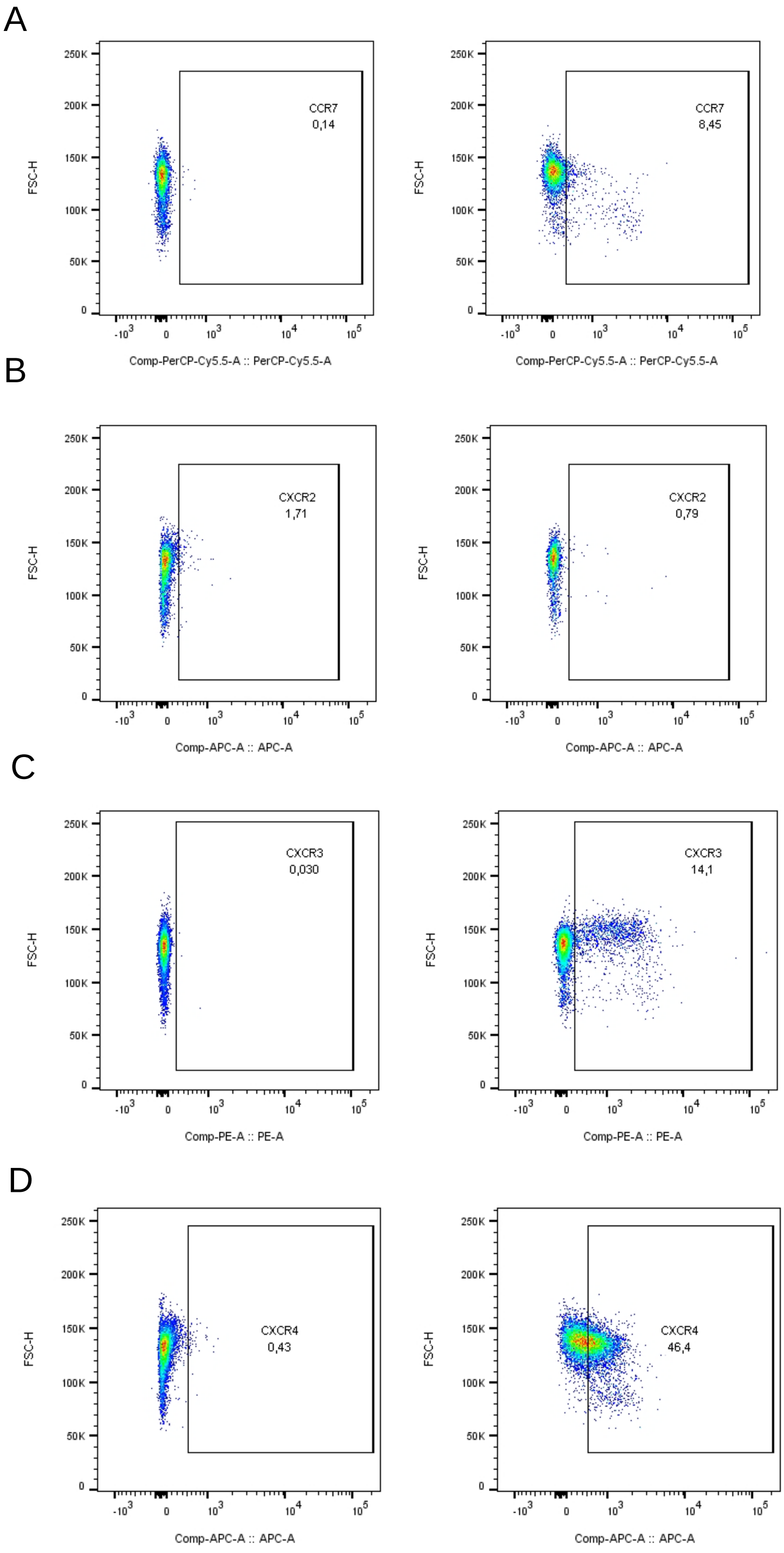

Supplementary figure 5. Chemokine receptor expression on spleen CD8+ T-cells in uninfected mice (black bars, d0) and HSV-2 infected mice (grey bars, d8). Data is expressed as percentage of CD8+ T-cells expressing CCR2, CCR4, CCR5, CCR6, CCR7, CXCR2, CXCR3 or CXCR4. Statistical analysis was performed using ANOVA with Tukeys multiple comparisons test, where **** indicates $p<0.0001$ and *** $p < 0.001$ using GraphPad Prism version 7 (GraphPad Software).

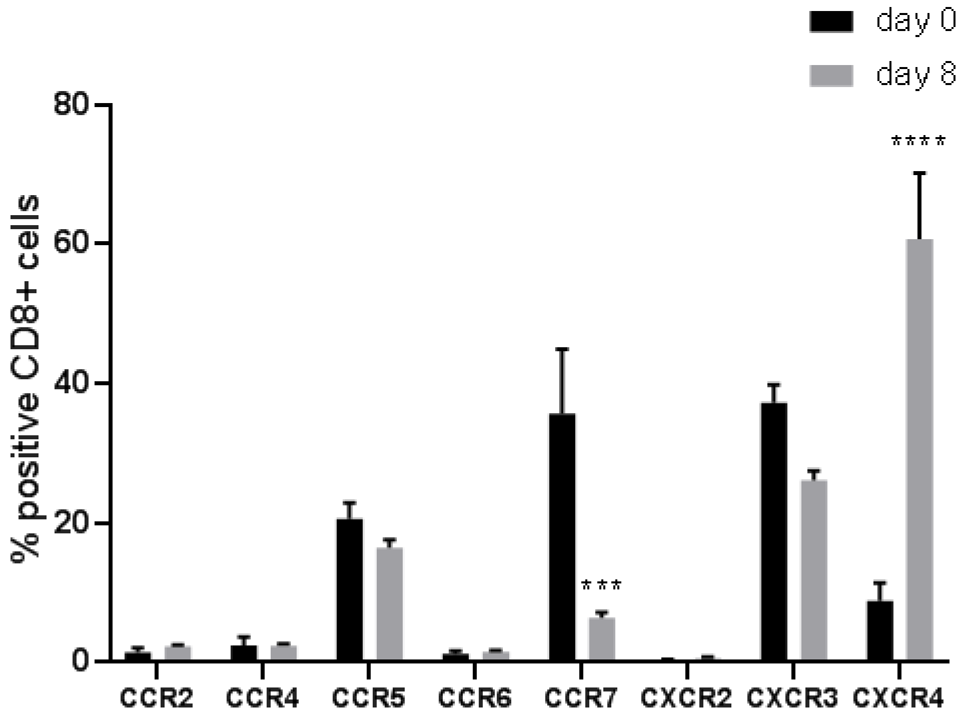

Supplement: Supplementary file 1 — Supplementary file1 (PDF 627 KB) [file 13365_2020_940_MOESM1_ESM.pdf]
